# Long-Term Efficacy and Safety of Adhesion Prevention Agents in Abdominal and Pelvic Surgeries: A Systematic Review

**DOI:** 10.7759/cureus.71280

**Published:** 2024-10-11

**Authors:** Sergio Rodrigo Oliveira Souza Lima, Kimberly Kanemitsu, Muhammad Rashid, Vaishvik K Patel, Muhammad Ali

**Affiliations:** 1 Plastic Surgery, Hospital da Bahia, Salvador, BRA; 2 Clinical Sciences, Windsor University School of Medicine, Chicago, USA; 3 General Surgery, Allama Iqbal Medical College, Lahore, PAK; 4 School of Medicine, St. George's University, West Indies, GRD; 5 General Surgery, Nishtar Medical University, Multan, PAK

**Keywords:** abdominal surgery, adhesion barriers, adhesion prevention, bioresorbable membranes, hyaluronic acid-carboxymethylcellulose, icodextrin solution, pelvic surgery, postoperative adhesions, randomized controlled trials, small bowel obstruction

## Abstract

This systematic review evaluates the long-term efficacy and safety of adhesion prevention agents in abdominal and pelvic surgeries, synthesizing data from randomized controlled trials and meta-analyses. Adhesions, common postoperative complications, can lead to significant morbidity, including chronic pain, infertility, and bowel obstruction. Various agents, including hyaluronic acid-carboxymethylcellulose films and icodextrin solutions, have been developed to mitigate these risks. Our review highlights that agents like bioresorbable membranes (Seprafilm) and icodextrin significantly reduce the incidence and severity of adhesions, particularly in high-risk surgeries. However, certain complications such as anastomotic leaks and infections are associated with some agents, emphasizing the need for careful consideration in clinical decision-making. Additionally, while these agents reduce postoperative morbidity and enhance recovery, further research is needed to assess their long-term impact, particularly regarding fertility outcomes and chronic pain. This review underscores the importance of integrating adhesion prevention agents into surgical protocols, which has the potential to reduce healthcare costs, improve patient outcomes, and optimize postoperative care pathways. Standardization of adhesion prevention practices could further enhance surgical efficiency and patient recovery, particularly in high-risk patient populations and complex surgeries.

## Introduction and background

Postoperative adhesions are a prevalent and troublesome outcome of abdominal and pelvic surgeries, presenting a significant challenge in postoperative care and patient recovery [1]. These fibrous bands, which can form between any surfaces disrupted during surgical procedures, are implicated in a range of long-term complications such as chronic pain, bowel obstruction, and infertility [2]. The pathogenesis of adhesions involves a complex interplay of tissue ischemia, inflammation, and fibrin deposition, which, if not resolved by fibrinolytic activity, results in fibrous scar tissue. Historically, the prevention of adhesions has centered on surgical techniques that minimize tissue disruption and contamination. However, the intrinsic nature of certain surgeries and the variability in individual healing responses necessitate additional preventive measures [3].

In response to this clinical need, a variety of pharmacological and mechanical barrier agents have been developed and tested, including hyaluronic acid-carboxymethylcellulose films, icodextrin solutions, and other novel bioresorbable materials [4]. These products aim to create a physical or chemical separation between tissues during the critical period of postoperative healing. Their application ranges broadly from general abdominal procedures to specific gynecological and colorectal surgeries, where the risk of adhesion formation is notably high [5]. Despite widespread use, the efficacy and safety profiles of these adhesion barriers vary significantly across different surgical modalities and patient populations. This variability is reflected in the literature, where studies report conflicting results regarding their actual effectiveness in clinical practice [6].

The primary objective of this systematic review is to comprehensively evaluate the long-term efficacy and safety of adhesion prevention agents utilized during abdominal and pelvic surgeries. By synthesizing data from randomized controlled trials and meta-analyses, this review aims to ascertain the impact of these agents on reducing the incidence, severity, and subsequent clinical complications of postoperative adhesions. Moreover, the review seeks to identify any potential adverse effects associated with these agents, thereby guiding clinical practice and informing future research in the field of surgical care. The findings are intended to aid in the optimization of surgical outcomes and contribute to the body of knowledge on postoperative adhesion prevention, enhancing both patient outcomes and healthcare efficiency.

## Review

Materials and methods

Search Strategy

Our search strategy was carefully crafted following the Preferred Reporting Items for Systematic Reviews and Meta-Analyses (PRISMA) guidelines [7] to identify studies assessing the efficacy and safety of adhesion prevention agents in abdominal and pelvic surgeries. A thorough search was conducted across several major databases, including PubMed, MEDLINE, Embase, the Cochrane Library, and Scopus. The search covered literature from the inception of each database up to September 2024, ensuring a comprehensive review of both historical and the most current data.

Keywords and Medical Subject Headings (MeSH) terms were specifically chosen to align closely with our research question, involving combinations such as "adhesion prevention," "abdominal surgery," "pelvic surgery," "bioresorbable barriers," "hyaluronic acid," "carboxymethylcellulose," and "randomized controlled trials." These terms were used with Boolean operators ('AND', 'OR') to formulate precise search strings, for example, "adhesion prevention AND abdominal surgery AND randomized controlled trials" and "pelvic surgery AND bioresorbable barriers AND efficacy." To enhance the scope of our search, we also reviewed the reference lists of all retrieved articles for additional relevant studies and searched clinical trial registries and conference proceedings to identify unpublished or ongoing research in this field.

This search strategy was exhaustive and was peer-reviewed by an information specialist to ensure the comprehensiveness and accuracy of the search process, adhering strictly to systematic review standards for identifying all pertinent studies regarding adhesion prevention in surgical settings.

Eligibility Criteria

The eligibility criteria for this systematic review were rigorously specified to ensure the inclusion of studies that provided robust and pertinent data on the effectiveness and safety of adhesion prevention agents in abdominal and pelvic surgeries. We included peer-reviewed research articles such as randomized controlled trials and clinical trials that examined the use of various adhesion barriers like hyaluronic acid-carboxymethylcellulose films, icodextrin solutions, and other innovative materials intended to prevent postoperative adhesions. These studies involved patients undergoing abdominal or pelvic surgical procedures where adhesion prevention agents were utilized either during or post-surgery.

Inclusion criteria were comprehensive to ensure that only studies meeting high methodological standards were considered. This included studies that provided clear data on the incidence, severity, and potential complications associated with adhesions following the use of these agents. All selected studies were published in English, in peer-reviewed journals, and the search covered literature from the inception of the databases up to the current date to ensure that the most recent and relevant findings were included. Conversely, exclusion criteria omitted studies that did not focus specifically on human subjects undergoing abdominal or pelvic surgeries, those that did not assess the specified adhesion prevention agents, or those that lacked rigorous outcome reporting. Additionally, non-peer-reviewed articles, grey literature, and studies published in languages other than English were excluded to maintain a high standard of data reliability and to streamline the synthesis process.

Data Extraction

The data extraction process for our systematic review on the efficacy and safety of adhesion prevention agents in abdominal and pelvic surgeries is rigorously structured to ensure high precision and validity of the gathered information. Initially, two independent reviewers will conduct a preliminary screening of articles by assessing titles and abstracts to determine their relevance based on predefined criteria, categorizing them as "relevant," "not relevant," or "potentially relevant." This stage is crucial to narrow down the focus to the most pertinent studies.

Subsequently, articles classified as potentially relevant undergo a detailed full-text review to ascertain their eligibility for inclusion in the review. We utilize a custom-designed data extraction form, developed in a digital spreadsheet tool, to standardize the collection of vital data across all studies. This form is meticulously completed by each reviewer independently and includes fields for extracting data on study characteristics such as the lead author, year of publication, study design, sample size, main outcomes, type of adhesion prevention agent used, and key findings, along with any reported complications or adverse effects. In cases of disagreement between the reviewers, a consensus is sought through discussion, or if necessary, a third reviewer is consulted to make a final determination. This systematic approach ensures that the data extraction process is both thorough and consistent, laying a robust foundation for subsequent analysis and synthesis of the findings relevant to the use of adhesion prevention agents in surgical settings.

Data Analysis and Synthesis

Given the diverse methodologies and outcomes across the selected studies, we employed a qualitative approach for data analysis and synthesis rather than conducting a formal meta-analysis. This approach allowed us to systematically assess the effectiveness and safety of various adhesion prevention agents used in abdominal and pelvic surgeries. We categorized the studies based on key variables such as the type of adhesion barrier used, surgical context, and reported outcomes, including incidence, severity, and complications related to adhesions. By grouping studies with similar interventions and analyzing trends, we were able to identify recurring themes and variations in efficacy across different patient populations and surgical procedures. This narrative synthesis enabled us to evaluate the clinical relevance and applicability of the interventions, assess the strength of the evidence, and explore any gaps in the existing literature, thereby offering a comprehensive overview of the current state of adhesion prevention research.

Results

Study Selection Process

**Figure 1:**
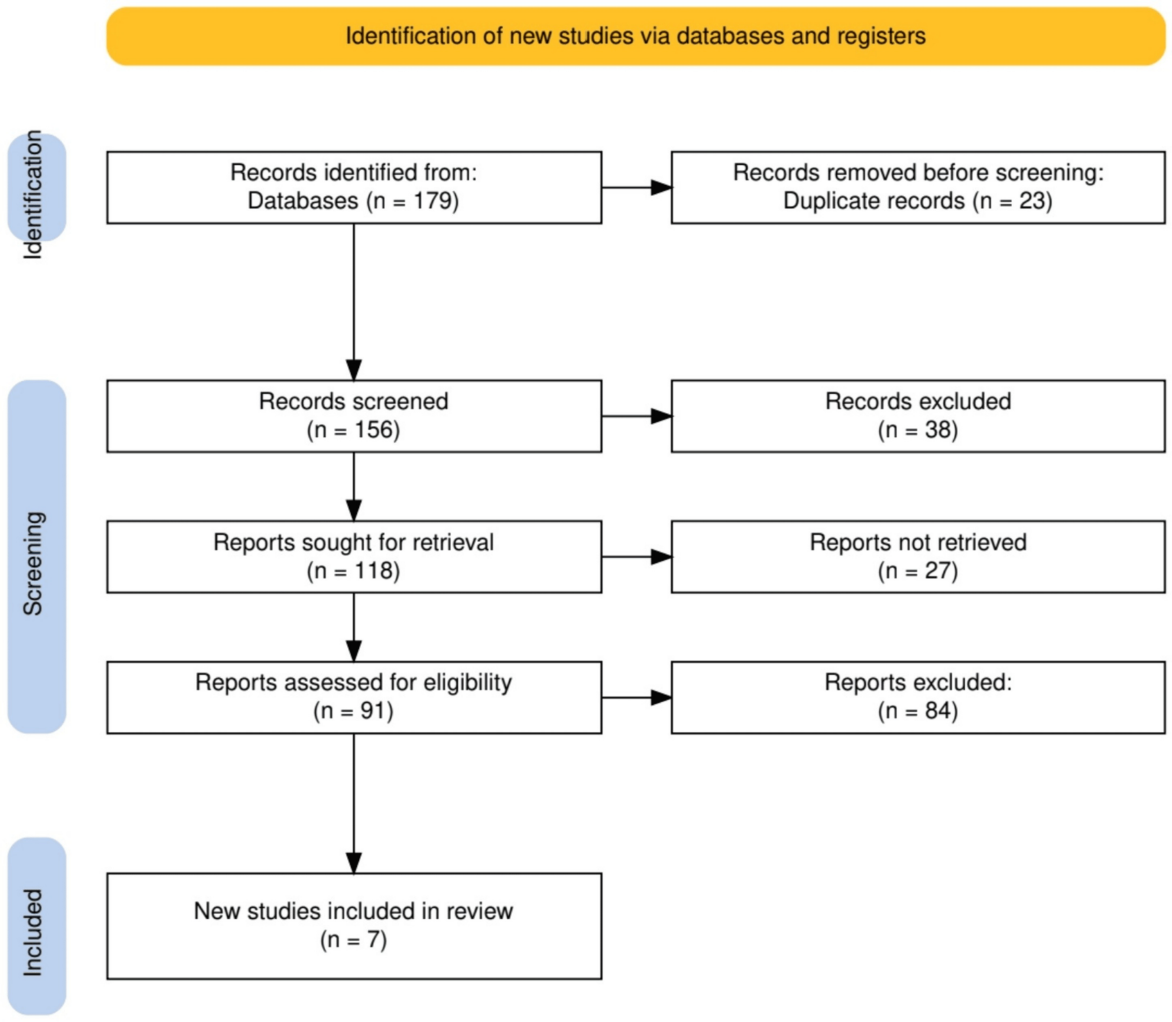
The PRISMA flowchart represents the study selection process. PRISMA: Preferred Reporting Items for Systematic Reviews and Meta-Analyses

The study selection process began with identifying 179 records from database searches, with 23 duplicates removed prior to screening. A total of 156 records were screened based on title and abstract, leading to the exclusion of 38 irrelevant studies. Of the remaining 118 reports sought for retrieval, 27 were not retrieved. Consequently, 91 reports were assessed for eligibility, but 84 were excluded due to not meeting the inclusion criteria. Ultimately, seven new studies were included in the systematic review. This rigorous process ensured that only the most relevant and high-quality studies were selected for comprehensive analysis. The study selection process is given in Figure 1.

Characteristics of the Selected Studies

The selected studies in this review represent a diverse range of methodologies, objectives, and outcomes related to adhesion prevention agents in abdominal and pelvic surgeries. These studies include meta-analyses and randomized controlled trials, assessing the efficacy and safety of various agents such as hydroflotation, gels, resorbable collagen membranes, and icodextrin solutions. The studies span a variety of surgical settings, from gynecological and colorectal surgeries to cases of adhesive small bowel obstruction. Key findings across these studies indicate that adhesion prevention agents generally reduce the incidence and severity of adhesions, although some agents, such as Seprafilm, were associated with an increased risk of complications like anastomotic leaks. The quality of evidence varies, but most studies offer strong support for the use of these agents in improving surgical outcomes, with particular attention to reducing postoperative complications and enhancing patient recovery. A summary of the key studies is given in Table 1.

**Table 1:** Summary of key studies evaluating adhesion prevention agents in abdominal and pelvic surgeries. RCT: randomized controlled trial; GRADE: Grading of Recommendations, Assessment, Development, and Evaluations; ASBO: adhesive small bowel obstruction; p-values: probability values (statistical significance)

Lead author	Objective	Methods	Main findings	Quality of evidence	Conclusions
Ahmad et al., 2020 [8]	Evaluate the effectiveness and safety of fluid and pharmacological agents on rates of pain, live births, and adhesion prevention in women undergoing gynecological surgery.	Meta-analysis of 32 RCTs, assessed using Cochrane standard methodological procedures and GRADE.	Hydroflotation agents may reduce adhesion incidence, but there are unclear effects on pelvic pain and live births. Gel agents reduced adhesion incidence, but effects on mean adhesion scores and clinical pregnancy rates were uncertain. Steroids showed unclear effects on live birth and clinical pregnancy rates.	Varied from very low to high, depending on the outcome and agent studied.	Gels and hydroflotation agents are effective for adhesion prevention. There is no evidence supporting improvement in fertility outcomes or pelvic pain. Further research is needed.
Stommel et al., 2014 [9]	Assess the efficacy and safety of C-Qur™ Film in decreasing the incidence of adhesions after colorectal surgery.	Prospective, investigator-initiated, randomized, double-blinded, multicenter trial. Patients undergoing colorectal resection with temporary ostomy were randomized to treatment with C-Qur™ Film or no adhesion barrier.	The study hypothesizes that using C-Qur™ Film under the primary incision could reduce the incidence of adhesions by 30%. Patients will be evaluated during ostomy takedown surgery for adhesion incidence, extent, and severity.	Good, supported by the rigorous design of a randomized, double-blinded, multicenter trial with adequate sample size and methodology, ensuring reliable and generalizable results.	The study will add to the evidence on the use of anti-adhesive barriers in colorectal surgery. The incidence of adhesions at the incision site is chosen as the primary outcome due to its potential implications on clinical complications.
Dabrowski et al., 2016 [10]	Assess the efficacy and safety of COVA+™, a resorbable collagen membrane, for preventing postoperative adhesions in abdominal surgery.	Prospective multicenter study of 113 patients undergoing either bariatric surgery or reversal of a diverting stoma. Patients were divided into two groups: those treated with a collagen membrane and a control group without it.	The use of COVA+™ resulted in better outcomes compared to the control group in terms of incidence, severity, and extent of adhesions. A significant reduction in operative duration and complications was observed when COVA+™ was used.	Results suggest a statistically significant benefit, with reported p-values indicating strong evidence for the findings (p<0.001 and p<0.005 for different measurements).	COVA+™ effectively prevents postoperative adhesions, lowering their incidence, severity, and extent. It is safe to use and facilitates easier reoperations.
Hajibandeh et al., 2022 [11]	Evaluate the effect of hyaluronate-based bioresorbable membrane (Seprafilm) on outcomes of abdominal surgery, specifically focusing on its efficacy in preventing adhesions and other complications.	Meta-analysis and trial sequential analysis of 13 RCTs involving 3665 patients comparing outcomes with and without Seprafilm.	Seprafilm significantly reduced the risk of small bowel obstruction and severity of adhesions but increased the risk of anastomotic leak. There were no significant differences in surgical site infections, intra-abdominal abscess, or ileus.	High, with trial sequential analysis confirming the conclusiveness of the meta-analysis findings.	Seprafilm effectively reduces the risk of small bowel obstruction and the severity of adhesions after abdominal surgery but is associated with an increased risk of anastomotic leak. It is recommended for use in non-anastomosis surgeries.
Cohen et al., 2005 [12]	Assess the safety and efficacy of a glycerol/sodium hyaluronate/carboxymethylcellulose-based bioresorbable membrane in reducing the incidence, extent, and severity of postoperative adhesions in abdominal surgery.	Prospective, randomized, evaluator-blinded multicenter study. One hundred and twenty patients undergoing restorative proctocolectomy with ileal pouch-anal anastomosis were randomized to treatment with the membrane or no anti-adhesion treatment.	Treatment significantly reduced the incidence, extent, and severity of adhesions compared to control. Thirty-three percent of treated patients had no adhesions versus 10% in the control. A higher incidence of abscess and wound complications was noted.	Significant findings supported by p-values (p=0.002 and p<0.001 for different measures).	The membrane effectively reduced adhesions but was associated with an increased risk of infection-related complications. The safety profile was similar to the control group, except for the noted complications.
Catena et al., 2012 [13]	Evaluate the safety and effectiveness of icodextrin 4% solution in reducing the incidence, extent, and severity of adhesions in patients after abdominal surgery for ASBO.	Prospective, randomized, controlled, single-center study comparing icodextrin 4% solution to no anti-adhesion treatment in patients undergoing laparotomy for ASBO. Primary endpoints include ASBO recurrence and adhesion formation.	The use of icodextrin 4% solution significantly reduced the ASBO recurrence rate compared to the control (2.19% vs. 11.11%, p<0.05). There were no significant differences in the need for repeat laparotomies. Adhesion severity was lower in the icodextrin group, although not statistically significant.	Statistically significant findings for recurrence rates, with p<0.05 indicating strong evidence.	Icodextrin 4% solution is safe and effectively reduces the risk of intra-abdominal adhesion formation and subsequent ASBO recurrence.
van der Wal et al., 2011 [14]	Determine the long-term effects of hyaluronic acid-carboxymethylcellulose membrane (Seprafilm) on the incidence of adhesions, small bowel obstruction, and chronic abdominal complaints after colorectal surgery (Hartmann's procedure).	Randomized clinical trial comparing the intraperitoneal placement of Seprafilm to a control group without it in patients undergoing Hartmann's procedure. Follow-up included direct visual evaluation and patient questionnaires.	Seprafilm did not reduce the incidence of small bowel obstruction but significantly decreased the incidence of chronic abdominal complaints compared to controls (35.3% vs. 77.8%, p=0.018).	Evidence includes significant statistical findings for chronic abdominal complaints with a p-value of 0.018, suggesting a strong evidence level for this specific outcome.	While Seprafilm did not prevent small bowel obstruction, it effectively reduced chronic abdominal complaints after Hartmann's procedure.

Discussion

Our systematic review evaluated several adhesion prevention agents used in abdominal and pelvic surgeries across a range of studies. One of the most consistent findings was the effectiveness of bioresorbable membranes, such as hyaluronic acid-carboxymethylcellulose (Seprafilm), and icodextrin solutions in reducing the incidence and severity of adhesions. Studies like those by Hajibandeh et al. [11] and Cohen et al. [12] demonstrated that Seprafilm significantly reduced small bowel obstruction risk and chronic abdominal pain, although an increased risk of anastomotic leaks and infection-related complications was noted. Similarly, icodextrin 4% solution, as evaluated by Catena et al. [13], proved effective in reducing adhesion recurrence rates in patients undergoing surgery for adhesive small bowel obstruction. C-Qur™ Film and COVA+™ collagen membranes were also shown to provide measurable reductions in adhesion formation and operative complications in colorectal surgeries, as reported by Stommel et al. [9] and Dabrowski et al. [10].

Notably, several studies identified conflicting results regarding adhesion prevention agents' impact on secondary outcomes like pelvic pain and fertility. For example, Ahmad et al. [8] highlighted that while hydroflotation and gel agents reduced adhesion incidence, they had unclear effects on pelvic pain and clinical pregnancy rates. Overall, the findings underscore the value of adhesion barriers in surgical practice, but they also point to the need for further high-quality studies to assess their long-term impacts on broader clinical outcomes, particularly fertility and the prevention of other postoperative complications. These insights contribute significantly to understanding the utility of adhesion prevention agents in enhancing postoperative recovery and patient outcomes.

Our findings largely align with the existing body of literature that supports the efficacy of adhesion prevention agents, particularly bioresorbable barriers like Seprafilm and icodextrin solutions [15]. Multiple studies, such as those by ten Broek et al. [16] and Menzies et al. [17], similarly highlighted the reduction in postoperative adhesions and small bowel obstructions with these agents. The consistency between our review and prior literature reinforces the utility of adhesion barriers in abdominal and pelvic surgeries. However, a notable difference emerged in the reported rates of complications, such as anastomotic leaks and wound infections, particularly with Seprafilm [18]. For example, our review identified an increased incidence of anastomotic leaks with Seprafilm use [11], which is less frequently reported in older studies [19]. This discrepancy could be attributed to differences in study populations, surgical techniques, or improved reporting standards in more recent trials.

Variations in results across studies may also stem from the heterogeneity in study designs and population characteristics. For instance, while some studies focused on high-risk patients undergoing complex surgeries such as colorectal resections, others, like those involving gynecological procedures such as those defined by Ahmad et al. [8], demonstrated less conclusive results, particularly in outcomes like fertility and pelvic pain. The type of surgical procedure, the skill level of the surgeons, and the inherent biological differences between patient populations, such as age, comorbidities, or the type of surgery, can contribute to these observed variations. Furthermore, inconsistencies in outcome measures, such as the assessment of adhesion severity and follow-up periods, further complicate direct comparisons. These factors underscore the need for standardization in clinical trials investigating adhesion prevention to provide more definitive guidance on their use in specific surgical contexts.

The findings of this systematic review provide valuable insights for clinicians and surgeons in guiding the use of adhesion prevention agents during abdominal and pelvic surgeries. Given the demonstrated efficacy of bioresorbable membranes like Seprafilm and COVA+™ and solutions such as icodextrin 4%, these agents should be considered an essential component of postoperative care, particularly in high-risk surgeries where adhesions can lead to significant complications like small bowel obstruction and chronic pain [3]. Incorporating these agents into clinical practice can help minimize postoperative morbidity, reduce the need for reoperations, and ultimately improve patient outcomes. Additionally, as seen with agents like Seprafilm, careful consideration of their risks, such as anastomotic leaks, should inform surgical decision-making, especially in cases where anastomoses are involved [20]. Surgeons must weigh the benefits of reduced adhesion formation against potential complications, tailoring the choice of adhesion prevention to each patient's risk profile and surgical context.

These findings may influence clinical guidelines by encouraging the inclusion of specific adhesion prevention agents in protocols for surgeries known to carry a high risk of postoperative adhesions. For healthcare systems, the use of these agents could potentially reduce long-term costs associated with adhesion-related complications, including hospital readmissions, prolonged recovery times, and additional surgical interventions [21]. The incorporation of these agents into routine practice can lead to a significant reduction in patients suffering from complications like bowel obstruction, as well as improved quality of life for patients undergoing repeat surgeries. Additionally, standardizing the use of adhesion prevention agents in appropriate cases could lead to optimized postoperative care pathways, enhancing the efficiency of surgical departments and improving overall healthcare outcomes [22].

A key strength of this systematic review lies in the comprehensive search strategy employed, which included multiple databases and sources to ensure thorough coverage of relevant literature. Rigorous inclusion criteria were applied, focusing exclusively on randomized controlled trials and high-quality meta-analyses, which provided a robust and reliable dataset. Additionally, the systematic approach to data extraction and synthesis allowed for a detailed and consistent analysis of the efficacy and safety of adhesion prevention agents. However, there are limitations to consider. The heterogeneity among the included studies, particularly in terms of study design, patient populations, and surgical techniques, introduces variability that may affect the interpretation of results. Furthermore, some studies lacked long-term follow-up, limiting the ability to assess the sustained efficacy of these agents over time. Publication bias, where positive results are more likely to be published than negative ones, may also have influenced the findings. These factors suggest that while the conclusions are supported by strong evidence, caution should be exercised when generalizing the results to all surgical settings and patient populations.

This review highlights several gaps in the current literature, particularly the need for more randomized controlled trials with extended follow-up periods to better assess the long-term efficacy and safety of adhesion prevention agents. While many studies demonstrate short-term benefits, their long-term impact, especially regarding fertility outcomes and chronic pain, remains unclear. Future research should focus on novel adhesion prevention agents that may offer improved safety profiles, particularly in reducing the risk of complications like anastomotic leaks [23]. Additionally, certain patient populations, such as those with pre-existing conditions like diabetes or immunocompromised individuals, have not been adequately studied in the context of adhesion prevention. Research into these subgroups could yield valuable insights into tailored approaches for different patient demographics. Further innovation in surgical techniques, such as minimally invasive approaches combined with adhesion barriers, may also enhance the effectiveness of adhesion prevention strategies and optimize postoperative recovery.

## Conclusions

This systematic review demonstrates that adhesion prevention agents, particularly bioresorbable membranes and barrier solutions, can significantly reduce the incidence and severity of postoperative adhesions in abdominal and pelvic surgeries. However, despite their widespread use, there is variability in their effectiveness, with certain agents associated with adverse events like anastomotic leaks and infection-related complications. The review confirms that while these agents contribute to better surgical outcomes in high-risk procedures, further long-term studies are needed to fully assess their impact on critical outcomes such as fertility and chronic pain, which remain underexplored. These findings emphasize the importance of individualized patient care when selecting adhesion prevention agents, as their risks and benefits vary across surgical contexts and patient populations.

A key knowledge gap identified through this review is the lack of standardized guidelines for the use of these agents, particularly in complex surgeries with varying risks of adhesion formation. Additionally, there is insufficient long-term data evaluating their impact on broader clinical outcomes such as quality of life and the need for reoperations. Future research should focus on these areas, with a particular emphasis on optimizing the balance between adhesion prevention and minimizing complications like infection and anastomotic failure. Addressing these gaps could lead to more consistent and safe use of adhesion prevention agents in clinical practice, ultimately improving patient recovery and reducing healthcare costs associated with postoperative complications.
